# Canadian Association of Gastroenterology Clinical Practice Guideline for Immunizations in Patients With Inflammatory Bowel Disease (IBD)—Part 1: Live Vaccines

**DOI:** 10.1093/jcag/gwab015

**Published:** 2021-07-29

**Authors:** Eric I Benchimol, Frances Tse, Matthew W Carroll, Jennifer C deBruyn, Shelly A McNeil, Anne Pham-Huy, Cynthia H Seow, Lisa L Barrett, Talat Bessissow, Nicholas Carman, Gil Y Melmed, Otto G Vanderkooi, John K Marshall, Jennifer L Jones

**Affiliations:** 1 Department of Pediatrics and School of Epidemiology and Public Health, University of Ottawa, Ottawa, Ontario, Canada; 2 CHEO Inflammatory Bowel Disease Centre, Division of Gastroenterology, Hepatology and Nutrition, Children’s Hospital of Eastern Ontario, and CHEO Research Institute, Ottawa, Ontario, Canada; 3 Department of Paediatrics, University of Toronto, Toronto, Ontario, Canada, SickKids Inflammatory Bowel Disease Centre, Division of Gastroenterology Hepatology and Nutrition, The Hospital for Sick Children, Child Health Evaluative Sciences, SickKids Research Institute, ICES, Toronto, Ontario, Canada; 4 Division of Gastroenterology and Farncombe Family Digestive Health Research Institute, McMaster University, Hamilton, Ontario, Canada; 5 Division of Pediatric Gastroenterology, Hepatology and Nutrition, Department of Pediatrics, University of Alberta, Edmonton, Alberta, Canada; 6 Section of Pediatric Gastroenterology, Departments of Pediatrics and Community Health Sciences, University of Calgary, Calgary, Alberta, Canada; 7 Division of Infectious Diseases, Department of Medicine, Dalhousie University, Halifax, Nova Scotia, Canada; 8 Division of Infectious Diseases, Immunology and Allergy, Department of Pediatrics, Children’s Hospital of Eastern Ontario, University of Ottawa, Ottawa, Ontario; 9 Division of Gastroenterology, Departments of Medicine and Community Health Sciences, University of Calgary, Calgary, Alberta, Canada; 10 Division of Gastroenterology, McGill University Health Centre, Montreal, Quebec, Canada; 11 Department of Pediatrics, University of Ottawa, Ottawa, Ontario, Canada; 12 CHEO Inflammatory Bowel Disease Centre, Division of Gastroenterology, Hepatology and Nutrition, Children’s Hospital of Eastern Ontario, Ottawa, Ontario, Canada; 13 Inflammatory Bowel Disease Center, Cedars-Sinai Medical Center, Los Angeles, California; 14 Section of Infectious Diseases, Departments of Pediatrics, Microbiology, Immunology and Infectious Diseases, Pathology and Laboratory Medicine and Community Health Sciences, University of Calgary, Alberta Children’s Hospital Research Institute, Calgary, Alberta, Canada; 15 Department of Medicine and Community Health and Epidemiology, Dalhousie University, Queen Elizabeth II Health Sciences Center, Halifax, Nova Scotia, Canada

## Abstract

**Background & Aims:**

Patients with inflammatory bowel disease (IBD) may be at increased risk of some vaccine-preventable diseases. The effectiveness and safety of vaccinations may be altered by immunosuppressive therapies or IBD itself. These recommendations, developed by the Canadian Association of Gastroenterology and endorsed by the American Gastroenterological Association, aim to provide guidance on immunizations in patients with inflammatory bowel disease. This publication focused on live vaccines.

**Methods:**

Systematic reviews evaluating the efficacy, effectiveness, and safety of vaccines in patients with IBD, other immune-mediated inflammatory diseases, and the general population were performed. Critical outcomes included mortality, vaccine-preventable diseases, and serious adverse events. Immunogenicity was considered a surrogate outcome for vaccine efficacy. Certainty of evidence and strength of recommendations were rated according to the GRADE (Grading of Recommendation Assessment, Development, and Evaluation) approach. Key questions were developed through an iterative process and voted on by a multidisciplinary panel. Recommendations were formulated using the Evidence-to-Decision framework. Strong recommendation means that most patients should receive the recommended course of action, whereas a conditional recommendation means that different choices will be appropriate for different patients.

**Results:**

Three good practice statements included reviewing a patient’s vaccination status at diagnosis and at regular intervals, giving appropriate vaccinations as soon as possible, and not delaying urgently needed immunosuppressive therapy to provide vaccinations. There are 4 recommendations on the use of live vaccines. Measles, mumps, rubella vaccine is recommended for both adult and pediatric patients with IBD not on immunosuppressive therapy, but not for those using immunosuppressive medications (conditional). Varicella vaccine is recommended for pediatric patients with IBD not on immunosuppressive therapy, but not for those using immunosuppressive medications (conditional). For adults, recommendations are conditionally in favor of varicella vaccine for those not on immunosuppressive therapy, and against for those on therapy. No recommendation was made regarding the use of live vaccines in infants born to mothers using biologics because the desirable and undesirable effects were closely balanced and the evidence was insufficient.

**Conclusions:**

Maintaining appropriate vaccination status in patients with IBD is critical to optimize patient outcomes. In general, live vaccines are recommended in patients not on immunosuppressive therapy, but not for those using immunosuppressive medications. Additional studies are needed to evaluate the safety and efficacy of live vaccines in patients on immunosuppressive therapy.

Patients with inflammatory bowel disease (IBD) may be at increased risk of some vaccine-preventable infections, but vaccination coverage remains low (1). Primary care providers often do not feel comfortable vaccinating patients with IBD (2), and gastroenterologists may assume that vaccination is the responsibility of primary care providers (3).

The effectiveness, safety, and appropriateness of vaccinations can be altered in patients with IBD due to the underlying immune dysregulation inherent to IBD and/or requirement for immunosuppressive therapy (ie, corticosteroids, thiopurines, biologics, small molecules such as JAK inhibitors, and combinations thereof), which can impair immune responses (4,5). In addition, there are concerns about potential adverse effects related to live vaccines. Live vaccines may cause disease by uncontrolled viral replication. These include measles, mumps, rubella; rotavirus; smallpox; chickenpox; yellow fever; and Bacillus Calmette-Guérin (BCG) vaccines.

Previous guidelines on immunizations of patients with IBD considered only the limited available evidence of vaccine safety and effectiveness in IBD populations, and failed to consider the ample evidence available in the general population or in other immune-mediated inflammatory diseases when assessing the certainty of evidence (CoE) or developing their recommendations (6,7). Most of the recommendations were conditional based on low or very low level of evidence (6). Therefore, a new guideline based on a comprehensive systematic review and assessment of the CoE of the benefits and harms of immunizations in patients with IBD, and considering all available evidence in the general population and other immune-mediated inflammatory diseases, will help guide best practice and enhance decision-making.

Existing systematic reviews, evidence, and guidelines in the general population were used as an evidence base, where appropriate, and assessed in conjunction with available data in the IBD population. General population guidelines referred to include those from the Centers for Disease Control and Prevention (CDC)–Advisory Committee on Immunization Practices (ACIP) (8), World Health Organization (WHO) (9), and National Advisory Committee on Immunization (NACI), and the Public Health Agency of Canada Canadian Immunization Guide (10).

These guidelines were developed in the context of the low prevalence of vaccine-preventable infections in North America and Europe, and it is recognized that more aggressive steps may be needed during outbreaks or in high-prevalence areas. Vaccination of patients with the rare infantile-onset form of IBD was not discussed and, in such cases, clinicians should consult with an immunologist and infectious diseases specialist.

These evidence-based recommendations developed by the Canadian Association of Gastroenterology and endorsed by the American Gastroenterological Association, aim to provide guidance on immunizations in patients with inflammatory bowel disease. This publication is the first of two articles and focuses on live vaccines; part 2 is focused on inactivated vaccines (11). These recommendations, developed by the Canadian Association of Gastroenterology and endorsed by the American Gastroenterological Association, aim to provide guidance on immunizations in patients with inflammatory bowel disease.

## Methods

The guideline panel assessed the certainty of the supporting evidence and developed the recommendations following the GRADE (Grading of Recommendation Assessment, Development, and Evaluation) approach (12). The overall guideline development process, including panel formation, management of conflicts of interests, internal and external review, and organization approval, was guided by Canadian Association of Gastroenterology (CAG) policies and procedures derived from the Guideline International Network-McMaster Guideline Development Checklist (https://cebgrade.mcmaster.ca/guidelinechecklistonline.html) and was intended to meet the standards for trustworthy guidelines by the Institute of Medicine and the Guideline International Network (13,14).

### Scope and Purpose

This guideline focuses on common vaccine-preventable diseases (VPDs) and live vaccines and is inclusive of both adult and pediatric (birth through 18 years) populations with IBD in North America and Europe. The recommendations are not meant to be extrapolated to special patient subgroups or special situations (eg, infantile-onset or monogenic forms of IBD and travelers). The target audience for this guideline includes health care providers, policy-makers, and patients with IBD. Please note that both the live attenuated herpes zoster (HZ) vaccine and recombinant HZ vaccine will be addressed in the article focusing on inactivated vaccines because the recombinant vaccine has supplanted the live vaccine as the preferred choice (11).

### PICO Development

PICO (patient population, intervention, comparator, and outcome) questions were developed by the steering committee and methodologists, and finalized through a consensus process of iterative discussions with all other voting participants.

For each vaccine, the patient population was divided into adult and pediatric subgroups a priori. For certain vaccines, the patient populations were further subdivided, depending on likely disease burden from VPDs, including age-specific mortality and morbidity and CoE in the safety and effectiveness of vaccines. Critical outcomes were determined a priori to include mortality, VPDs, and serious adverse events. Immunogenicity was considered a surrogate outcome that may be important for decision-making.

### Systematic Synthesis of the Literature

#### Literature searches

Systematic searches of the published English-language literature including MEDLINE, EMBASE, Cochrane Database of Systematic Reviews, and Cochrane Central Register of Controlled Trials (all via OVIDSP) from 1989 through April 12, 2019, were conducted by the Cochrane Gut Group at McMaster University. Studies evaluating the efficacy, effectiveness, and safety of vaccines in patients with IBD were included as direct evidence. When there was paucity of evidence in the IBD population, indirect evidence in other immune-mediated inflammatory diseases was sought. As well, a systematic search for systematic reviews and meta-analyses in the general population was conducted for each vaccine. When available, the CDC–ACIP (8) and the WHO (9) GRADE evidence profile tables in the general population were reviewed and incorporated into the overall GRADE assessment. Literature searches for studies assessing the baseline risk of VPDs in patients with IBD were conducted for each vaccine to inform decision-making. In addition, literature reviews focused on patients’ values and preferences and cost-effectiveness related to vaccines in patients with IBD when available and in the general population were also conducted.

Key search terms and search strategies are available in Appendix 2. Two methodologists (F.T., M.C.) performed duplicate screening of literature search results, data extraction, and risk of bias assessment of the primary studies. Existing systematic reviews were used as a baseline source where appropriate and updated or improved as needed.

#### Assessment of the certainty of evidence

Methodologists (F.T., M.C.) used the GRADE approach to assess the CoE for each PICO question (12). For each vaccine, the evidence of its safety and effectiveness in the general population was used as an anchor. In some cases, the CoE for effectiveness was downgraded for indirectness when there was evidence suggesting that the vaccines may be less immunogenic or effective in IBD populations. However, if there were studies done in IBD populations that supported the findings of effectiveness in the general population, the evidence was not downgraded. In most cases, the CoE for safety was downgraded because small sample sizes of IBD studies could not detect rare adverse events. The full methods are presented in detail in Appendix 3. Methodologists (F.T., M.C.) prepared evidence profile tables for each PICO question (Appendix 3), which were provided along with the supporting literature to members of the group before the consensus meeting.

### Moving From Evidence to Recommendations

A face-to-face meeting was held in Ottawa, Ontario, Canada in October 2019. The voting members of the consensus group included 11 adult and pediatric gastroenterologists, infectious diseases specialists, and vaccinologists from Canada and the United States. Also in attendance were the 2 methodologists (F.T., M.C.), a moderator (J.K.M.), and representatives from the CAG. Participants with direct conflicts of interest with vaccine manufacturing companies participated in the discussion, but did not vote on PICO questions (Appendix 1). Three patient/patient advocates were involved in the process, including providing feedback on the PICO questions and reviewing the manuscript. Finally, the recommendations were reviewed, commented on, and endorsed by the American Gastroenterological Association.

At the consensus meeting, the methodologists presented evidence for each of the PICO questions, and the GRADE Evidence-to-Decision framework was applied to develop recommendations based on the CoE; the balance of benefits and harms, patients’ values and preferences; resource implications; acceptability; and feasibility (Appendix 3) (15–17). After discussion of the PICO questions, voting members anonymously indicated the direction of recommendation with yes, no, or uncertain/neutral. Consensus was met when ≥75% of votes were for a specific direction (either yes or no). When consensus was reached for a PICO question, a second anonymous vote on the strength of recommendation (strong or conditional) was conducted. A level of agreement of ≥75% of participants was needed to support a “strong” recommendation, and the phrase “we recommend” would be used. If this threshold was not met, the recommendation defaulted to “conditional.” Where there was low or very low CoE, the strength of the recommendation would default to “conditional,” and the phrase “we suggest” would be used. As per the GRADE method, a strong recommendation means that the panel is very confident that the benefits of following the recommendation clearly outweigh the harms (or vice versa), so the course of action should apply to most patients (Tables 1 and 2) (12,18). A conditional recommendation is one for which the panel concludes that the desirable effects of adherence to a recommendation probably outweigh the undesirable effects, but the panel is not confident about these tradeoffs due to low or very low CoE, uncertainty regarding the balance of benefits and harms, uncertainty or variability in patients’ values and preferences, or questionable cost-effectiveness. Thus, conditional recommendations mandate shared decision-making.

**Table 1:** Certainty of Evidence and Definitions (12)

Certainty of evidence	Definition
High	Further research is very unlikely to change our confidence in the estimate of effect
Moderate	Further research is likely to have an important impact on our confidence in the estimate of effect and may change the estimate
Low	Further research is very likely to have an important impact on our confidence in the estimate of effect and is likely to change the estimate
Very low	Any estimate of effect is very uncertain

**Table 2:** Interpretation of Strong and Conditional Recommendations (18)

Implications	Strong recommendation	Conditional recommendation
For patients	Most individuals in this situation would want the recommended course of action and only a small proportion would not	Most individuals in this situation would want the suggested course of action, but many would not
For clinicians	Most individuals should receive the recommended course of action	Different choices will be appropriate for different individuals consistent with the patient’s values and preferences. Use shared decision-making
For policy makers	The recommendation can be adopted as policy in most situations	Policy-making will require substantial debate and involvement of various stakeholders

NOTE. Strong recommendations use “we recommend,” and conditional recommendations use “we suggest.”

For PICO questions for which the group failed to reach consensus, recommendations were not developed; however, summaries of the relevant evidence and discussions are provided. Three recommendations were determined to be “good practice statements” (19), with the consensus group agreeing that there is high level of certainty that the recommendations will have unequivocal net benefits based on a large body of indirect evidence, but may not be widely recognized or used.

The manuscript was initially drafted by the co-chairs and methodologists, followed by dissemination to the remaining members of the consensus group for review and approval. As per CAG policy for all clinical practice guidelines, the manuscript was made available to all CAG members for commenting before submission. In addition, the manuscript was reviewed by 2 patient/patient advocates to obtain feedback on the clarity, acceptability, and importance of the document. Finally, the recommendations were reviewed, commented on, and endorsed by the American Gastroenterological Association.

### Role of the Funding Sources

This guideline was supported through unrestricted grants to CAG by the Canadian Institutes of Health Research Institute of Nutrition, Metabolism and Diabetes, and CANImmunize, which had no involvement in any aspect of the guideline development or manuscript preparation.

## Principles of Immunization of Patients With Inflammatory Bowel Disease

The individual recommendation statements are provided and include the strength of recommendation, CoE, and voting result. This is followed by a discussion of the evidence considered for the specific recommendation. A summary of the recommendations is provided in Table 3. See Appendix 3 for the evidence profile tables with detailed CoE assessments (including description of study limitations, inconsistency, indirectness, imprecision, and publication bias) and summary of findings, and the Evidence-to-Decision frameworks.

**Table 3:** Summary of Consensus Recommendations for Immunizations in Patients With Inflammatory Bowel Disease

Principles of immunization of patients with IBD
Recommendation 1: In all patients with IBD, a complete review of the patient’s history of immunization and VPDs should be performed at diagnosis and updated at regular intervals by IBD care providers. Ungraded good practice statement.
Recommendation 2: In patients with IBD, all appropriate vaccinations should be given as soon as possible, and ideally prior to initiation of immunosuppressive therapy. Ungraded good practice statement.
Recommendation 3: In patients with IBD who require urgent immunosuppressive therapy, treatment should not be delayed in order to provide vaccinations. Ungraded good practice statement.
Live vaccines
MMR
Recommendation 4A: In MMR-susceptible pediatric patients with IBD not on immunosuppressive therapy, we recommend MMR vaccine be given. GRADE: Strong recommendation, moderate CoE Recommendation 4B: In MMR-susceptible pediatric patients with IBD on immunosuppressive therapy, we suggest against giving MMR vaccine. GRADE: Conditional recommendation, very low CoE
Recommendation 5A: In MMR-susceptible adult patients with IBD not on immunosuppressive therapy, we recommend MMR vaccine be given. GRADE: Strong recommendation, moderate CoE Recommendation 5B: In MMR-susceptible adult patients with IBD on immunosuppressive therapy, we suggest against giving MMR vaccine. GRADE: Conditional recommendation, very low CoE
Varicella
Recommendation 6A: In varicella-susceptible pediatric patients with IBD not on immunosuppressive therapy, we recommend varicella vaccine be given. GRADE: Strong recommendation, moderate CoE Recommendation 6B: In varicella-susceptible pediatric patients with IBD on immunosuppressive therapy, we suggest against giving varicella vaccine. GRADE: Conditional recommendation, very low CoE
Recommendation 7A: In varicella-susceptible adult patients with IBD not on immunosuppressive therapy, we suggest varicella vaccine be given. GRADE: Conditional recommendation, very low CoE Recommendation 7B: In varicella-susceptible adult patients with IBD on immunosuppressive therapy, we suggest against giving varicella vaccine. GRADE: Conditional recommendation, very low CoE
Statements with no recommendations
No Recommendation A: In infants born of mothers using biologic therapies, the consensus group could not make a recommendation for or against giving live vaccines in the first 6 months of life.

CoE, certainty of evidence; MMR, measles-mumps-rubella; VPDs, vaccine preventable diseases.

***Recommendation 1: In all patients with IBD, a complete review of the patient’s history of immunization and vaccine preventable diseases should be performed at diagnosis and updated at regular intervals by IBD care providers***.

Ungraded good practice statement.

***Recommendation 2: In patients with IBD, all appropriate vaccinations should be given as soon as possible, and ideally prior to initiation of immunosuppressive therapy.***

Ungraded good practice statement.

***Recommendation 3: In patients with IBD who require urgent immunosuppressive therapy, treatment should not be delayed in order to provide vaccinations.***

Ungraded good practice statement.

Patients with IBD remain suboptimally immunized, potentially as a result of insufficient counseling from providers and patients’ lack of awareness and concerns about adverse events or lack of benefit (3,20,21), The importance of IBD care providers monitoring patient immunization status is emphasized by 1 report that found only 30% of primary care providers were comfortable vaccinating patients with IBD (2). despite care to IBD patients frequently being provided by family physicians, pediatricians, and nurse practitioners (22,23). Other studies have demonstrated that provider recommendation is the strongest predictor for receipt of preventative health services, including vaccination (3,24). Therefore, IBD care providers should take an active role in obtaining a vaccination history, providing recommendations to the primary care clinician for the appropriate vaccines to be administered, and assuring that their patients are appropriately immunized (25).

Patients with IBD are not considered immunosuppressed at diagnosis, but subsequently may become immunosuppressed due to IBD therapies. Observational studies have shown that IBD patients on immunosuppressive therapies have a significantly lower serologic response to routine vaccinations (26). Therefore, the ideal time to review a patient’s immunization status is at diagnosis. Furthermore, because vaccination recommendations vary by age, and the use of immunosuppressive therapies may change throughout a patient’s disease course, regular follow-up is necessary. Because patients may not always be aware of their immunization status, serologic testing may be useful when considering certain vaccines.

While in clinical practice, it may not always be practical to include a detailed vaccination history at every visit, the consensus group identified important time points that may prompt immunization review. These included medication changes that influence degree of immunosuppression, a change in risk factors for VPDs (eg, occupational risks or travel), when patients are due for an age-appropriate scheduled vaccine, and annually for vaccines, such as influenza. Incorporating reminders and checklists for vaccination into electronic medical records can be a useful strategy to increase vaccination uptake, ensure completion of vaccination schedules, and improve quality of vaccination services (27).

Ideally, immunizations for VPDs should be provided at a time with maximum benefits and expected immunogenicity, along with minimum adverse effects. For patients with IBD, this time should be before starting immunosuppressive therapy. There is no standard definition of immunosuppression. The degree to which immunosuppressive therapy causes clinically significant immunosuppression generally is dose-related and varies by drug. The CDC considers immunosuppressive therapy equivalent to ≥2 mg/kg/d or 20 mg/d of prednisone for ≥14 days as sufficiently immunosuppressive to raise concern about the safety of immunization with live vaccines (8). The Infectious Diseases Society of America defines low-level immunosuppression as prednisone <2 mg/kg (maximum of ≤20 mg/d); methotrexate ≤0.4 mg/kg/wk; azathioprine ≤3 mg/kg/d; or 6-mercaptopurine ≤1.5 mg/kg/d. High-level immunosuppression includes treatment with doses higher than those listed for low-level and biologic agents (28). Ultimately, the degree of immunosuppression for each patient is determined by the treating provider (8). The recommended timing for vaccinations before initiating immunosuppressive therapy in both the CDC and the NACI guidelines is at least 14 days for inactivated vaccines to optimize immunogenicity, and at least 4 weeks for live vaccines to additionally minimize the risk of vaccine-related disease (8,10). Live vaccines should not be administered for at least 3 months after immunosuppressive therapy (8,10). However, for patients who do require urgent immunosuppressive therapy, treatment should not be delayed in order to administer vaccines, because this could lead to more anticipated harms than benefits, due to the risk of progression of the inflammatory activity and resulting complications.

## Live Vaccines in Patients With Inflammatory Bowel Disease

### Measles, Mumps, Rubella

#### Risk of measles, mumps, rubella in people with inflammatory bowel disease compared to people without inflammatory bowel disease. Key evidence

The literature search did not identify any study on the risk of measles, mumps and rubella (MMR) in patients with IBD.

**Recommendation 4A: In MMR-susceptible pediatric patients with IBD not on immunosuppressive therapy, we recommend MMR vaccine be given.**

GRADE: Strong recommendation, moderate CoE. Vote on PICO question: yes, 100%

***Recommendation 4B: In MMR-susceptible pediatric patients with IBD on immunosuppressive therapy, we suggest against giving MMR vaccine.***

GRADE: Conditional recommendation, very low CoE. Vote on PICO question: no, 100%

#### Key evidence

In the setting of routine vaccination of pediatric patients, NACI defines “MMR-susceptible” as individuals who do not have a history of documented vaccination, laboratory-confirmed infection, or laboratory evidence of immunity (10). In healthy children, a WHO assessment of evidence for MMR vaccines rated the CoE as high for efficacy (9). The vaccine effectiveness was ≥95% for prevention of measles, 69%–81% for mumps, and ≥90% for rubella (9,29).

A systematic review of observational studies in patients with immune-mediated diseases included 2852 patients with IBD on immunosuppressive therapies (30). When comparing patients on and not on immunosuppressive therapy, the results were inconsistent, with some studies showing a reduced serological response and others showing no significant differences (30). Four observational studies assessed the serologic status of MMR in patients with IBD on immunosuppressive therapy (31–34). In the pediatric study, serologic protection rates were: 67.6% for measles, 63.3% for mumps, and 81.4% for rubella (32). In an adult study, there was no difference in antibody concentrations between those with IBD who received MMR vaccines as children prior to the diagnosis of IBD compared to healthy controls (31). However, the relevance of MMR serology is unknown, because MMR serology may be falsely negative despite previous vaccination (8).

In the WHO assessment of evidence, the CoE for safety in healthy children was rated as moderate (9). In a systematic review of healthy children up to 15 years of age, MMR vaccine was associated with a lower incidence of upper respiratory tract infections and a similar incidence of other adverse events compared to placebo (29). In the systematic review of studies in patients with immune-mediated diseases (including patients with IBD), most studies found that the use of live vaccines was safe in patients on immunosuppressive therapy (including prednisone 2.5–35 mg/d, methotrexate 5–27 mg/wk, 6-mercaptopurine, biologic monotherapy, and combination therapy with biologics and immunomodulators) (30). Serious adverse events (0.05%) and infections related to MMR vaccines (0.2%) were rare. Most infections were mild, but rare fatal cases have been reported with other live vaccines, such as BCG vaccine (35).

The CoE for effectiveness was anchored to the general population and was downgraded from high to moderate due to indirectness because data suggested reduced immunogenicity in pediatric patients with IBD. The CoE for safety was not downgraded from moderate, when applied to pediatric patients with IBD not on immunosuppressive medications. However, it was downgraded to very low due to indirectness when applied to patients on immunosuppressive therapy.

#### Discussion

Both NACI and the CDC recommend routine childhood vaccination against MMR, and catch-up vaccination for most previously unimmunized individuals (8,10). MMR vaccines are generally not recommended in individuals with impaired immune function.

An economic analysis showed that a MMR vaccination program in the US was cost-saving from both the direct and societal perspectives compared with no MMR vaccination (36). Although rate of uptake of childhood vaccines is generally high, one of the most common reasons cited for postponing or abstaining from MMR vaccination was fear of side effects (37–39).

Based on the data for efficacy and safety, the consensus group recommended that MMR-susceptible pediatric patients with IBD who are not on immunosuppressive therapy, be given the MMR vaccine.

For patients on immunosuppressive therapy, both CDC and NACI recommend assessing the degree of immunosuppression (8,10). They recommend against live vaccines in patients on immunosuppressive therapy equivalent to ≥2 mg/kg/d or 20 mg/d of prednisone for ≥14 days (8,10). The consensus group suggested against the MMR vaccine in pediatric patients with IBD on immunosuppressive therapy. However, because there is little evidence to define differential levels of immunosuppression, the group considered this to include all patients on such therapies. This was a conditional suggestion, and in the event of an outbreak or in a region that has a low-prevalence of immunization, patients should be referred to an infectious disease specialist for risk–benefit assessment.

***Recommendation 5A: In MMR-susceptible adult patients with IBD not on immunosuppressive therapy, we recommend MMR vaccine be given.***

GRADE: Strong recommendation, moderate CoE. Vote on PICO question: yes, 100%

***Recommendation 5B: In MMR-susceptible adult patients with IBD on immunosuppressive therapy, we suggest against giving MMR vaccine.***

GRADE: Conditional recommendation, very low CoE. Vote on PICO question: no, 100%

#### Key evidence

There are sparse data on MMR vaccine administered outside the standard childhood schedule. One randomized controlled trial comparing 2 MMR vaccines included mainly healthy adults who had received at least 1 previous dose of MMR vaccine, and found sero-response rates of >98% (40). The safety and immunogenicity data in adults mirror the findings in pediatric populations; therefore, the evidence was not downgraded for indirectness. As in the pediatric population, there is moderate CoE that MMR vaccines are effective in adults with IBD. There is moderate CoE that MMR vaccines are safe in adults with IBD not on immunosuppressive therapy, but very low CoE of safety in those on immunosuppressive therapy.

#### Discussion

Both NACI and the CDC recommend MMR vaccines only for susceptible adults at high risk, including health care workers, military personnel, post-secondary students, and individuals traveling outside North America (8,10). No cost-effectiveness data in adults were found.

The consensus group concluded that susceptible adults with IBD may be at high risk, and recommended vaccination for those not on immunosuppressive therapy. As in pediatric patients, the group suggested against the vaccine in those on immunosuppressive therapy because of safety concerns.

### Varicella

#### Risk of varicella in people with inflammatory bowel disease compared to people without inflammatory bowel disease. Key evidence:

In temperate countries, there is near-universal varicella zoster virus (VZV) seroconversion by late childhood (41,42). Primary VZV infection is often more severe in adults than in children (43). In contrast to primary VZV infection, reactivation of the VZV (HZ or shingles) tends to occur more frequently in older adults (ie, older than 50 years) and in those who are immunosuppressed (see recommendations 10A and 10B in part 2) (11,44–49). Reports of primary VZV infection in patients with IBD include cases of severe disease course and fatalities. In a review of 20 cases of primary VZV infection in patients with IBD on immunosuppressive therapy (16 adults and 4 children), there were 5 deaths (50). A retrospective cross-sectional inpatient study found a strong association between hospitalizations due to primary VZV and HZ and IBD in pediatric patients (51).

The CoE was downgraded from high to very low due to study limitations and indirectness. Patients with IBD may be more likely to be diagnosed and admitted due to VZV or HZ than non-IBD controls.

***Recommendation 6A: In varicella-susceptible pediatric patients with IBD not on immunosuppressive therapy, we recommend varicella vaccine be given.***

GRADE: Strong recommendation, moderate CoE. Vote on PICO question: yes, 100%

***Recommendation 6B: In varicella-susceptible pediatric patients with IBD on immunosuppressive therapy, we suggest against giving varicella vaccine.***

GRADE: Conditional recommendation, very low CoE. Vote on PICO question: no, 100%

#### Key evidence

In the setting of routine vaccination of pediatric patients, NACI defines “varicella-susceptible” as individuals who do not have documented immunization with 2 doses of a varicella-containing vaccine, or laboratory evidence of immunity (10). A WHO assessment of evidence for effectiveness of varicella vaccines in healthy children included 3 systematic reviews (52). In addition, a more recent systematic review, including 42 observational studies, found the effectiveness in preventing varicella infection with 1-dose and 2-dose vaccines was 81% and 92%, respectively (53). In a systematic review of 40 observational studies in patients with immune-mediated diseases (including IBD), the seroconversion rates were high but appeared to be reduced by immunosuppressive therapy (30). In 2 cross-sectional studies, 70% of pediatric patients with IBD who had a history of varicella vaccination or chickenpox infection demonstrated serologic protection, but patients with past chickenpox infection mounted higher titers of varicella IgG than patients with varicella vaccination (32,54). Current immunosuppressive therapy was not associated with serologic protection (32). However, due to low test sensitivity to detect antibodies after vaccination, previously vaccinated individuals are likely to be immune to varicella, even if the antibody test is negative. Hence, CDC does not recommend serologic testing before or post immunization for varicella (8).

In the WHO assessment of evidence, the CoE for safety in healthy children was rated as moderate (9). In a systematic review of studies in patients with immune-mediated diseases (including 20,556 IBD patients) on immunosuppressive therapy, serious adverse events (0.05%) and infections related to VZV vaccines (1.0%) were rare (30). Most infections were mild, but rare fatal cases have been reported with other live vaccines. In a large safety analysis published outside of the literature review parameters, including data on more than 212 million doses of VZV vaccines, disseminated disease caused by the vaccine strain was confirmed in 39 cases (55). Of these, 28 occurred in patients on immunosuppressive therapies. No fatal infections have been reported after VZV vaccination in patients with IBD. However, a case of disseminated wild-type VZV infection was reported in a patient with IBD on immunosuppressive therapy (56).

The CoE was anchored to the general population. For effectiveness, the CoE was downgraded from high to moderate due to indirectness, as observational studies suggested the vaccines may be less immunogenic in patients with IBD. The CoE for safety was not downgraded from moderate when applied to pediatric patients with IBD not on immunosuppressive medications, but was downgraded to very low due to indirectness when applied to patients on immunosuppressive therapy.

**Discussion:** Both NACI and the CDC recommend routine childhood VZV vaccination (8,10). An economic analysis in the United States found the universal VZV program to be cost-saving from the societal perspective compared with no vaccination (57). Since introduction of the universal VZV vaccination program, there has been a dramatic decline in the incidence of varicella, but also increased rates of HZ (58,59). Debate continues as to whether universal VZV vaccination program leads to unintended increase in HZ incidence in the short term due to the theory of VZV vaccination limiting the exogenous boosting of immunity to HZ and, therefore, whether universal VZV vaccination is cost-effective, given the potential increase in morbidity associated with HZ. Patient acceptability of the vaccine is impacted by insufficient information about the vaccine, fear of adverse effects, preference of natural illness, and financial limitations (60).

Based on the evidence, the consensus group recommended VZV vaccination for varicella-susceptible pediatric patients with IBD who are not on immunosuppressive therapy. Manufacturers of varicella-containing vaccines recommend avoidance of medications derived from salicylic acid, such as mesalamine, medication and Reye’s syndrome was discussed by the consensus group, no evidence exists of this association in children with IBD. Therefore, the consensus group did not recommend against VZV vaccination in children using mesalamine who received VZV vaccination.

Patients who are immunocompromised are more susceptible to infections and more likely to experience severe disease and complications (10). As is the case with MMR vaccine, NACI and ACIP generally recommend against live vaccines in people who are immunocompromised, with consideration of the degree of immunosuppression (8,10).

The consensus group suggested against the vaccine in patients on immunosuppressive therapy. Patients with IBD should be assessed for prior vaccination or exposure and susceptible patients should be vaccinated before initiating immunosuppressive therapy when possible. As recommended in guidelines for patients with immune-mediated disorders on immunosuppressive therapies, individual risks and benefits should be assessed (61), and the consensus group suggested that such patients be referred to an infectious disease specialist for assessment.

***Recommendation 7A: In varicella-susceptible adult patients with IBD not on immunosuppressive therapy, we suggest varicella vaccine be given.***

GRADE: Conditional recommendation, very low CoE. Vote on PICO question: yes, 100%

***Recommendation 7B: In varicella-susceptible adult patients with IBD on immunosuppressive therapy, we suggest against giving varicella vaccine.***

GRADE: Conditional recommendation, very low CoE. Vote on PICO question: no, 100%

#### Key evidence

In 4 observational studies of healthy adults considered susceptible to varicella infection, there was a 0.26%–7% rate of mild varicella infection after VZV vaccination (62–65). In 1 study, the vaccine efficacy rate was estimated to be 51% in healthy adults (64), in contrast to 92% in healthy children (53). However, seroconversion rates were high (92%–99%) and no serious adverse events were reported (62–65). Data for patients on immunosuppressive therapy were mainly in children, as described in recommendations 6A and 6B. The CoE for efficacy and safety was downgraded from low to very low due to indirectness and imprecision.

#### Discussion

Both NACI and the CDC recommend VZV vaccine for adults (younger than 50 years) without evidence of immunity (8,10). A cost-effectiveness analysis found that serologic testing of young adult immigrants to Canada without a self-reported history of varicella, followed by VZV vaccination of susceptible individuals would be a cost-saving approach compared to other strategies, such as no intervention, vaccination of all individuals, or serologic testing of all individuals and vaccination of those with results indicating susceptibility to varicella (66). Similarly, serologic testing of health care workers without a known history of varicella, followed by vaccination of susceptible individuals, was the most cost-effective strategy compared with no intervention (67).

Given the very low CoE, the consensus group made conditional suggestions in favor of vaccination for susceptible adults with IBD not on immunosuppressive therapy, and against vaccination for those on immunosuppressive therapy.

### Infants Born of Mothers Using Biologic Therapies

***No recommendation A (see Appendix 3, 13): In infants born of mothers using biologic therapies, the consensus group could not make a recommendation for or against giving live vaccines in the first 6 months of life.***

GRADE for PICO: very low CoE. Vote on PICO question: uncertain/neutral, 67%; no, 33%

#### Key evidence

The main concern around administering live vaccines to children born of mothers who have received biologic therapy is that of safety in the potentially immunocompromised newborn. Evidence suggests these infants can have detectable levels of certain monoclonal antibody biologic therapies at birth, with some drugs being detectable up to 12 months of age (68). Due to a lack of published evidence for other biologics, only anti-TNF biologics were discussed.

In North America, the live attenuated rotavirus vaccine is routinely given starting around 2 months of age. Other live vaccines, such as the MMR and varicella vaccine, are given at or after 12 months of age. Both NACI and ACIP recommend that the first dose of a rotavirus vaccine be given before 15 weeks of age, as the safety of providing the first dose of rotavirus vaccine in older infants is not known (8,10); therefore, the discussion around this recommendation was focused largely on the safety of the rotavirus vaccine.

In 7 observational studies (small cohort studies and case series) of infants exposed to biologic agents in utero, 56 infants received rotavirus vaccine at less than 6 months of age, 74 received BCG vaccine within 6 months of age, and 52 received MMR or rubella vaccine at 15 months of age (35,69–74). In most cases, the biologic therapy was stopped in the second or third trimester, and there were generally no serious adverse events among the infants. However, there was 1 death attributed to disseminated BCG infection after administration of the BCG vaccine to a 3-month old infant with in utero anti-tumor necrosis factor (anti-TNF) exposure (35).

Observational studies have reported detectable anti-TNF drug concentrations in cord blood at delivery even when the drugs were stopped in the second or third trimester (68,75,76). Detectable concentrations can persist for up to 6 months for adalimumab-exposed infants, and up to 12 months in infliximab-exposed infants (n = 1 of 80) after birth (68). The mean time to drug clearance in infants was 4.0 months (95% confidence interval, 2.9–5.0 months) for adalimumab and 7.3 months (95% confidence interval, 6.2–8.3 months) for infliximab (68).

Immunophenotyping studies have shown that anti-TNF–exposed infants had more immature B- and helper T-cell phenotype at birth (73), which normalized by 12 months (73,77). A decreased response after mycobacterial challenge was noted in 1 study (73). Observational studies have found that infants exposed to anti-TNF in utero have appropriate response to inactivated vaccines with no serious adverse events (70–72,74,75,78).

Detectable anti-TNF levels and immunophenotyping in exposed infants are surrogate outcomes for immunosuppression, which in turn are surrogate outcomes for potential adverse events related to administration of live vaccines. The CoE for safety was downgraded to very low due to study limitations, indirectness, and imprecision.

#### Discussion

Live vaccines, including rotavirus, are not recommended in those with severe combined immunodeficiency or other significant immunocompromising conditions (8,10). The CAG recommendations for the management of IBD in pregnancy, recommended against administration of live vaccinations within the first 6 months of life for newborns of women who were on anti-TNF therapy during pregnancy (79). Although there was low CoE, the recommendation was strong, based on the potential for catastrophic harm associated with early use of live vaccines. If vaccinations are deemed necessary, measuring monoclonal antibody drug levels in the infant and performing immunologic testing may help inform decisions.

Cost-effectiveness analyses have found that routine rotavirus vaccination programs in high-income settings are generally not cost-effective from a health system perspective (80–83). This is largely related to the fact that the majority of rotavirus infections in developed countries do not require emergency department visits or hospital admission (80,83). However, in global economic evaluations, rotavirus vaccine programs were cost-effective in low- and middle-income settings, and conclusions varied between studies in developed countries (82,83). In surveys, rotavirus vaccine was generally acceptable to parents, but many did not perceive the disease to be an important health issue (84,85).

Evidence suggests that infants born of mothers using monoclonal antibody biologic therapies can have detectable drug levels up to 12 months of age (68). The main concern around administering live vaccines to children born of mothers who have received biologic therapy is their safety in the exposed newborn. Although a fatal event was reported after BCG vaccine (35), a cohort study published after our search date found a low risk of adverse events among 90 infants who were last exposed to anti-TNF agents before or during the third trimester, and there were no serious adverse events, such as tuberculosis or death (86). In addition, the BCG vaccine elicits an immune response (T-cell mediated and humoral (87)) that is very different from that seen with rotavirus (partial IgA-mediated and T-helper (88)) and MMR (antibody-mediated, with CD8 response (89)) vaccines. However, despite this mechanistic difference, the consensus group remained uncertain of the safety of live vaccines, given the evidence for detectable levels of biologic therapies in infants up to 12 months after birth. In this setting, the group was unable to recommend for or against their routine use because the desirable and undesirable effects were closely balanced and the evidence on safety outcomes was insufficient to justify a recommendation. The group also did not find evidence to support the safety and effectiveness of temporary or permanent suspension of biologics in the third trimester of pregnancy in order to give live vaccines to infants born of mothers using biologic therapies in the first 6 months of life. Health care providers should be cautious with the administration of live vaccines in the first year of life in the infants of mothers using biologics. These infants should be evaluated by clinicians with expertise in the impact of exposure to monoclonal antibody biologics in utero.

## Supplementary Material

Note: To access the supplementary material accompanying this article, visit the online version of Gastroenterology at www.gastrojournal.org, and at http://dx.doi.org/10.1053/j.gastro.2020.12.079.

gwab015_suppl_Supplementary_Appendix_1

gwab015_suppl_Supplementary_Appendix_2

gwab015_suppl_Supplementary_Appendix_3

## Abbreviations used in this paper

ACIP: Advisory Committee on Immunization Practices
anti-TNF: anti-tumor necrosis factor
BCG: Bacillus Calmette-Guérin
CAG: Canadian Association of Gastroenterology
CDC: Centers for Disease Control and Prevention
CoE: certainty of evidence
GRADE: Grading of Recommendation Assessment, Development and Evaluation
HZ: herpes zoster
IBD: inflammatory bowel disease
MMR: measles, mumps, rubella
NACI: National Advisory Committee on Immunization
VZV: varicella zoster virus
VPD: vaccine-preventable disease
WHO: World Health Organization

## Canadian Association of Gastroenterology Statement

This clinical practice guideline (CPG) on immunizations in patients with IBD was developed under the direction of Dr Eric I. Benchimol and Dr Jennifer L. Jones, in accordance with the policies and procedures of the Canadian Association of Gastroenterology (CAG) and under the direction of CAG Clinical Affairs. It has been reviewed by the CAG Clinical Affairs Committee and the CAG Board of Directors. The CPG was developed following a thorough consideration of medical literature and the best available evidence and clinical experience. It represents the consensus of a Canadian and US panel composed of experts on this topic. The CPG aims to provide a reasonable and practical approach to care for specialists and allied health professionals charged with the duty of providing optimal care to patients and families, and can be subject to change as scientific knowledge and technology advance and as practice patterns evolve. The CPG is not intended to be a substitute for physicians using their individual judgment in managing clinical care in consultation with the patient, with appropriate regard to all the individual circumstances of the patient, diagnostic and treatment options available, and available resources. Adherence to these recommendations will not necessarily produce successful outcomes in every case.
