# Supplementary material for: Canadian Association of Gastroenterology Clinical Practice Guideline for Immunizations in Patients With Inflammatory Bowel Disease (IBD)—Part 1: Live Vaccines
Source: J Can Assoc Gastroenterol. 2021 Jul 29;4(4):e59–71. doi: 10.1093/jcag/gwab015 (PMC8407487; doi:10.1093/jcag/gwab015)
Supplement: gwab015_suppl_Supplementary_Appendix_2 [file gwab015_suppl_Supplementary_Appendix_2.pdf]

APPENDIX 2: LITERATURE SEARCH STRATEGIES

Database: Embase <1974 to 2019 April 12>, OVID Medline Epub Ahead of Print, In-Process & Other Non-Indexed Citations, Ovid MEDLINE(R) Daily and Ovid MEDLINE(R) 1946 to Present, EBM Reviews - Cochrane Central Register of Controlled Trials <March 2019>, EBM Reviews - Cochrane Database of Systematic Reviews <2005 to April 10, 2019>
Search Strategy:
----
1 (Inflammatory bowel disease* or ulcerative colitis or Crohn* or IBD).tw,kw. (247708)
2 (vaccination* or vaccine* or vaccinated or active immunization*).tw,kw. (623184)
3 1 and 2 (2644)
4 limit 3 to yr="1989-Current" (2548)
5 limit 4 to english language [Limit not valid in CDSR; records were retained] (2429)
6 conference abstract.pt. or Congresses as Topic/ or Conference Review.pt. or "Journal: Conference Abstract".pt. (3625784)
7 5 not 6 (1771)
8 (exp animals/ or exp animal/ or exp nonhuman/ or exp animal experiment/ or animal model/ or animal tissue/ or non human/ or (rat or rats or mice or mouse or swine or porcine or murine or sheep or lambs or pigs or piglets or rabbit or rabbits or cat or cats or dog or dogs or cattle or bovine or monkey or monkeys or trout or marmoset$1 or basic research or cell lines or in vitro or animal model or canine).tw.) not (humans/ or human/ or human experiment/ or (human* or men or women or patients or subjects).tw.) (10414522)
9 7 not 8 (1369)
10 remove duplicates from 9 (771)

****

Supplement Search strategy for systematic review of vaccines in other immune mediated inflammatory diseases (rheumatoid arthritis, systematic lupus erythematosus, psoriasis, psoriatic arthritis) and in the general population in PubMed.

(((((((vaccina*[Title/Abstract] OR vaccine*[Title/Abstract] OR active immunization*[Title/Abstract]))) AND (((((((Arthritis, Rheumatoid[MeSH Terms]) OR rheumatoid arthritis[Title/Abstract])) OR ((Lupus Erythematosus, Systemic[MeSH Terms]) OR (systematic lupus erythematosus[Title/Abstract] OR SLE[Title/Abstract]))) OR ((Psoriasis[MeSH Terms]) OR Psoriasis[Title/Abstract])) OR ((Arthritis, Psoriatic[MeSH Terms]) OR psoriatic arthritis[Title/Abstract])) OR ((immune-mediated inflammatory disease*[Title] OR IMID[Title]))))) AND ((("systematic review"[Publication Type]) OR "meta analysis"[Publication Type]) OR (Systematic review[Title/Abstract] OR meta-analysis[Title/Abstract] OR Medline[Title/Abstract] OR Embase[Title/Abstract] OR Pubmed[Title/Abstract] OR Cochrane Central[Title/Abstract] OR literature search[Title/Abstract] OR literature review[Title/Abstract])))) AND english[Language]
